# Salivary Alpha-amylase Activity and Mild Cognitive Impairment Among Japanese Older Adults: The Toon Health Study

**DOI:** 10.14789/jmj.JMJ23-0013-OT

**Published:** 2023-06-22

**Authors:** NAHO YAMANE, AI IKEDA, KIYOHIDE TOMOOKA, ISAO SAITO, KOUTATSU MARUYAMA, ERI EGUCHI, KEIKO SUYAMA, AKIKO FUJII, TAMAMI SHIBA, KUMIKO TANAKA, AKIKO KOOKA, SATSUKI NAKAMURA, MASARU KAJITA, RYOICHI KAWAMURA, YASUNORI TAKATA, HARUHIKO OSAWA, ANDREW STEPTOE, TAKESHI TANIGAWA

**Affiliations:** 1 Department of Public Health, Juntendo University Faculty of Medicine, Tokyo, Japan; 1 Department of Public Health, Juntendo University Faculty of Medicine, Tokyo, Japan; 2 Sado General Hospital, Niigata, Japan; 2 Sado General Hospital, Niigata, Japan; 3 Department of Public Health and Epidemiology, Faculty of Medicine, Oita University, Oita, Japan; 3 Department of Public Health and Epidemiology, Faculty of Medicine, Oita University, Oita, Japan; 4 Laboratory of Community Health and Nutrition, Special Course of Food and Health Science, Department of Bioscience, Graduate School of Agriculture, Ehime University, Ehime, Japan; 4 Laboratory of Community Health and Nutrition, Special Course of Food and Health Science, Department of Bioscience, Graduate School of Agriculture, Ehime University, Ehime, Japan; 5 Department of Epidemiology, Fukushima Medical University School of Medicine, Fukushima, Japan; 5 Department of Epidemiology, Fukushima Medical University School of Medicine, Fukushima, Japan; 6 Department of Community Health Systems Nursing, Graduate School of Medicine, Ehime University, Ehime, Japan; 6 Department of Community Health Systems Nursing, Graduate School of Medicine, Ehime University, Ehime, Japan; 7 Faculty of Nursing, Shijonawate Gakuen University, Osaka, Japan; 7 Faculty of Nursing, Shijonawate Gakuen University, Osaka, Japan; 8 Department of Fundamental Nursing, Faculty of Life Sciences, Kumamoto University, Kumamoto, Japan; 8 Department of Fundamental Nursing, Faculty of Life Sciences, Kumamoto University, Kumamoto, Japan; 9 Department of Nursing, Faculty of Human Health and Welfare, St. Catherine University, Ehime, Japan; 9 Department of Nursing, Faculty of Human Health and Welfare, St. Catherine University, Ehime, Japan; 10 Department of Diabetes and Molecular Genetics, Graduate School of Medicine, Ehime University, Ehime, Japan; 10 Department of Diabetes and Molecular Genetics, Graduate School of Medicine, Ehime University, Ehime, Japan; 11 Department of Behavioural Science and Health, University College London, London, UK; 11 Department of Behavioural Science and Health, University College London, London, UK; 12 Department of Public Health, Juntendo University Graduate School of Medicine, Tokyo, Japan; 12 Department of Public Health, Juntendo University Graduate School of Medicine, Tokyo, Japan

**Keywords:** salivary alpha-amylase, mild cognitive impairment, psychological stress, cross-sectional study

## Commentary

Dementia is an important global issue, and early detection and intervention are critical. There is growing interest in identifying mild cognitive impairment (MCI) and intervening in patients’ lifestyle-related behaviors in clinical and community settings to prevent the progression of dementia^1-3)^. Previous studies have shown associations between psychological stress and cognitive decline^4)^, increased risk of developing MCI^5)^, and dementia^6)^. One of the human stress-response systems is the sympathetic-adrenal-medullary (SAM) axis^7, 8)^. Salivary alpha-amylase (sAA) is a biomarker of psychological stress, as it indicates activation of the SAM axis^9)^. Psychological stress is thought to increase β-adrenergic activity via activation of the SAM axis, which leads to an increase in sAA levels^9)^. Previous studies have further shown that elevated β-adrenergic activity leads to amyloid-β (Aβ) peptide production^10, 11)^, and Aβ peptide production and deposition play an important role in the pathogenesis of Alzheimer’s disease (AD)^12)^. We therefore hypothesized that sAA, an objective marker of the SAM axis, is associated with MCI via β-adrenergic activity, which would suggest that psychological stress contributes to cognitive decline. We conducted a large cross-sectional study to investigate this association in the elderly.

This cross-sectional study was a part of the Toon Health Study and our analysis involved 865 participants aged ≥ 65 years. Saliva samples were collected in the morning and the levels of salivary alpha-amylase were assayed. We evaluated MCI using the Japanese version of the Montreal Cognitive Assessment: a score of < 26 indicated MCI. In the statistical analysis, a multivariable-adjusted logistic regression analysis using sex-specific quartiles of sAA was performed to calculate the odds ratio (OR) and 95% confidence interval (CI) of MCI after adjusting for age, sex, drinking status, smoking status, body mass index, hypertension, diabetes mellitus, physical activity, education, social support, social network, and heart rate variability.

**Figure 1:**
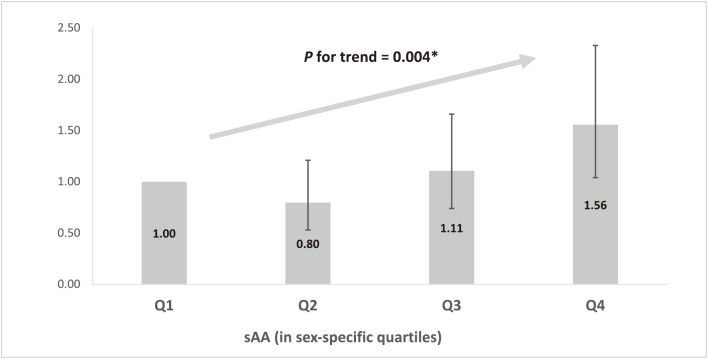
Association between MCI and sAA *P for trend associated 1SD increment of log-transformed sAA. Abbreviations: MCI, mild cognitive impairment; sAA, salivary alpha-amylase.

We found that sAA was significantly associated with MCI. The age- and sex-adjusted OR (95% CI) of MCI for the highest quartile group compared to the lowest was 1.56 (1.05-2.32). As shown in Figure 1, this significant association remained after adjusting for confounding factors. Moreover, the multivariable-adjusted OR (95% CI) for the 1-standard deviation increment of log-transformed sAA was 1.24 (1.07-1.44).

In summary, we found a significant association between sAA levels and MCI in elderly Japanese community dwellers. Our results are strongly consistent with previous studies that found associations between high levels of self-reported psychological distress and cognitive impairment^4)^ and dementia^6)^. Further, our results suggest that, since sAA is an objective marker of psychological stress, this stress contributes to cognitive decline. See the full article for further details: Yamane N. et al. Salivary Alpha-Amylase Activity and Mild Cognitive Impairment among Japanese Older Adults: The Toon Health Study. J Prev Alzheimers Dis 9, 752-757 (2022). https://doi.org/10.14283/jpad.2022.51.

## Funding

This study was supported by JSPS KAKENHI, under grant numbers JP16K09072, JP17KK0175, JP19K10670, and 25293142, and the 8020 Promotion Foundation, Japan in 2016 and 2017. The funders had no role in designing or conducting the study; in collecting, analyzing, or interpreting data; in preparing the manuscript; or in reviewing or approving the manuscript.

## Author contributions

TT and AI contributed to the study design. NY and AI analyzed and interpreted the data, and NY was the major contributor to writing the manuscript. All authors read and approved the final manuscript.

## Conflicts of interest statement

Dr. Ikeda reports grants from JSPS KAKENHI received during the study period. Dr. Saito reports grants received from the 8020 Promotion Foundation during the study period. Dr. Tanigawa reports grants received from JSPS KAKENHI during the study period. The remaining authors have nothing to disclose.
